# Insights Into Laparoscopic Port Site Complications: A Comprehensive Review

**DOI:** 10.7759/cureus.63431

**Published:** 2024-06-28

**Authors:** Nemi Chandra J, Sri Saran Manivasagam, Sushila Choudhary, Paras Manocha, B Harish Reddy

**Affiliations:** 1 General Surgery, Vardhman Mahavir Medical College and Safdarjung Hospital, New Delhi, IND; 2 Surgery, Maulana Azad Medical College, New Delhi, IND; 3 General Surgery, Sanjay Gandhi Memorial Hospital, New Delhi, IND

**Keywords:** laparoscopy, port site metastasis, port site hematoma, port site infection, port site hydatid cyst, port-site hernia

## Abstract

Laparoscopic surgery has become a widely accepted standard of care for numerous procedures in the modern world. Nearly every major surgical procedure previously only possible by employing open techniques may now be completed laparoscopically, attributable to the quick advancement of technology and surgeons' abilities. There are several complications associated with the laparoscopic port site, either infective, non-infective, or neoplastic. This study aims to explore the morbidity associated with the port site following laparoscopic surgery and discuss the risk factors for complications. The umbilical port was most frequently associated with port-site hernia (PSH), followed by the epigastrium and the left and right hypochondrium. Prolonged port manipulation and reinsertion, longer surgical times, failure to effectively close the fascial defect, and wound infection are responsible for the development of PSH. Port-site infection (PSI) is one avoidable adverse effect of laparoscopic surgery. Patients who have a history of diabetes, malnourishment, prolonged preoperative hospital stays, preoperative Staphylococcus aureus colonization of the nares, perioperative blood transfusions, and tobacco or steroid use are more likely to have PSI. Port-site hydatid cyst (PSHC) and port-site tuberculosis (PST) are rare but possible. While uncommon, a doctor should rule out endometriosis if a painful mass in the surgical scar, such as the trocar site, is discovered in a reproductive-age woman who has had pelvic or obstetric surgery in the past. Port-site metastasis (PSM) is the term for tumor-cell implantation at the trocar insertion site after a malignant tumor is removed laparoscopically. PSM has been reported in 1-2% of laparoscopic gynecologic surgical procedures. A few potential mechanisms for cell implantation at the port site include embolization of exfoliated cells during tumor dissection or hematogenous spread, air turbulence during long laparoscopic operations, and direct implantation onto the wound during forced, unprotected organ/tissue retrieval or from contaminated surgical instruments during tumor dissection. Nonetheless, the triggering mechanism is likely essentially multifaceted. Prevention is better than cure. Port-site hernia can be prevented using smaller trocars and meticulous rectus sheath defect closure at the end of surgery. The rest of the port site complications can be prevented by employing autoclavable laparoscopic hand instruments, utilizing autoclaved water to clean the instruments following disassembly, adhering to the recommended concentration, contact duration, and usage cycles when sterilizing instruments with liquid sterilizers, preventing bile or gut content from spilling into the operating room or the port site, using non-porous specimen retrieval bags for recovering the specimen, and thoroughly cleaning and irrigating the port site before closing the wound.

## Introduction and background

In the modern world, laparoscopic surgery has been widely accepted as a standard of care for numerous procedures. Nearly every major surgical procedure that was previously only possible employing open techniques may now be completed laparoscopically, attributable to the quick advancement of technology and the ability of surgeons [1]. Semm carried out the first laparoscopic appendectomy on September 13, 1980, at the University of Kiel's Department of Obstetrics and Gynecology. At that moment, it appeared to be a worldwide phenomenon and an unparalleled rarity. As a skilled doctor and gynecologist, Semm transformed the practice of conventional surgery. This invention happened so quickly that it had an immense impact, notably on gynecology. With the advent of laparoscopy in hysterectomy, urogynecological procedures, and oncological surgery, including lymphadenectomy in various physiological compartments, entirely new avenues could be explored. Laparoscopy also gained widespread acceptance in other areas, including urology and surgery [2]. On September 12, 1985, Prof. Dr. Med Erich Muhe of Boblingen, Germany, performed the first laparoscopic cholecystectomy (LC). France's Mouret carried out the first video-assisted laparoscopic cholecystectomy in 1987. This method was unanimously recognized as the standard of excellence for treating cholelithiasis in 1993. Following that time, fundoplications, appendectomies, splenectomies, nephrectomies, and a host of other procedures have been successfully completed using laparoscopy [3].

The tools and methods used in laparoscopic procedures have advanced significantly in recent years. There are several different trocar designs in use, and there are wide differences in views regarding fascial closure. The single-incision approach, sometimes referred to as single port access (SPA), single-incision laparoscopic surgery (SILS), single-site laparoscopy (SSL), or laparo-endoscopic single-site surgery (LESS), is one particular form that is rapidly coming into favor. Using either a larger trocar with several access channels or multiple trocars puncturing the fascia within the skin hole, the surgery is carried out through a single skin incision [4]. Following laparoscopic procedures, the most common complications or injuries are gastrointestinal (0.6 per 1,000), genitourinary (0.3 per 1000), vascular (0.1 per 1,000), and momentum (0.4 per 1,000). Pyoderma gangrenosum, metastases at the port site after laparoscopic oncosurgery, and port-site infections (PSIs) are among the less common side effects [5]. There are several complications associated with the laparoscopic port site, either infective, non-infective, or neoplastic. The goal of the current review was to gather the most recent data on potential problems related to the placement of a laparoscopic port.

## Review

Methods

Numerous complications are linked to the port placement site. The following keywords were used in our search for relevant publications published between 1996 and 2024: port-site hernia, port-site infection, port-site tuberculosis, port-site endometriosis, port-site hydatid cyst, and port-site metastases. The searches were conducted using the PubMed and Google Scholar databases. The results were limited to the English language. Since port site issues are uncommon, we have reviewed the available case reports, case series, systematic reviews, randomized control trials, and original articles currently available to analyze the subject comprehensively. Abstracts, letters to the editor, and comments were excluded. A total of 23 articles [4,6-27] were analyzed across the spectrum to prepare a comprehensive review and current consensus on this topic, as illustrated in Table 1.

**Table 1:** Brief description of included articles PSH: port-site hernia, DR: diastasis recti, PSI: port-site infection, PSHC: port-site hydatid cyst, PST: port-site tuberculosis, ATT: antitubercular therapy, PSE: port-site endometriosis, FNAC: fine-needle aspiration cytology.

S. no.	Author	Study design	Conclusion
1.	Bunting et al. [4]	A systematic review on PSH	The most critical factors for PSH were older age, higher body mass index, preexisting hernia, trocar design, diameter, increased surgery duration, and port-site extension for gallbladder extraction.
2.	Ki et al. [6]	Retrospective study on PSH	Port extension and sarcopenia were risk factors for umbilical PSH. DR might be a risk factor for umbilical PSH occurrence and recurrence.
3.	Nassar et al. [7]	Prospective observational study on PSH	Avoiding unnecessary wound extension and using non-absorbable sutures for defects greater than 2 cm, as well as in males with umbilical hernias, may help lower the incidence of incisional hernias.
4.	Calik et al. [8]	Randomized control study	Berci's needle closure of the umbilical port site has proved to be a safe and effective technique.
5.	Lambertz et al. [9]	Retrospective study on PSH	Port sites of a 10 mm or larger diameter fascia must be closed by suture. Depending on the size of the fascial defect, the patient's risk factors, and body mass index, port-site incisional hernias should be repaired with mesh or sutures.
6.	Tobe et al. [10]	Case report on PSH	After laparoscopic surgery, patients who present with minor intestinal blockage should have a port-site hernia assessed.
7.	Robicsek et al. [11]	Case report on PSH	One simple and efficient technique to lower the likelihood of port-site hernias is the fascial closure of port sites larger than 5 mm.
8.	Vineet et al. [12]	Case report on PSH	The rectus sheath must be closed for all ports with a diameter of 10 mm or more. When obese postoperative women experience persistent gastrointestinal symptoms after surgery, there needs to be a low threshold for obtaining cross-sectional imaging.
9.	Sasmal et al. [13]	A comprehensive review of PSI	The best way to prevent port-site infection is to strictly adhere to the laparoscopic equipment sterilization guidelines using the recommended sterilizing agent.
10.	Lilani et al. [14]	Prospective cohort study on PSI	A notable increase in the surgical site infection rate correlated with more prolonged surgical procedures. In patients with a drain compared to non-drained wounds (3.03%), the surgical site infection rate was significantly greater (22.41%).
11.	Mir et al. [15]	Prospective cohort study on PSI	Following an elective laparoscopic cholecystectomy, 6·7% of patients experience PSI. Pseudomonas is the bacterium that causes PSI most frequently.
12.	Aggarwal et al. [16]	Case report on PSHC	Port-site hydatid cysts are extremely rare. It happens due to scolices that lodge at the port site during a laparoscopy. Moreover, it could mimic a strangulated umbilical hernia, making the preoperative diagnosis difficult.
13.	Swamy et al. [17]	Case report on PSHC	Port-site Hydatid cysts can develop when scolices become lodged at the port site during the removal of a daughter cyst.
16.	Jagdish et al. [18]	Case report on PST	Complete surgical excision of the sinus tract is helpful.
14.	Cunnigaiper et al. [19]	Case report on PST	A biopsy of the chronic sinus at the port site helps arrive at a diagnosis. Healing by secondary intention is a viable option.
15.	Faridi et al. [20]	Case report on PST	A discharging sinus at the epigastric port site is a common presentation of PST, which responds to ATT.
17.	Emre et al. [21]	Case report on PSE	A doctor should rule out endometriosis if a painful mass in the port site is discovered in women who are fertile and have had prior pelvic or obstetric surgery.
18.	Siddiqui et al. [22]	Case report on PSE	When undergoing laparoscopic surgery, precautions should be followed to lower the risk of endometrial seeding. Remove all tissues and place them in the proper retrieval bag.
19.	Hensen et al. [23]	Retrospective case series on abdominal wall endometriosis (PSE)	Ultrasound-guided FNAC is a quick and precise diagnostic method.
20.	Grant et al. [24]	Retrospective study	In the context of endometrial cancer, isolated port-site metastases are linked to high rates of local control when treated with radiation and other multimodality therapies.
21.	Lago et al. [25]	Case-control study	If there are no macroscopic port-site metastases in patients undergoing laparoscopy before the debulking procedure, port site resection may not be advised.
22.	Agarwala et al. [26]	Retrospective cohort study	Patients with isolated port-site metastasis may benefit from a more aggressive therapy approach and careful patient selection.
23.	Z'graggen et al. [27]	Prospective cohort study	Recurrences at the port site are common in patients undergoing laparoscopic cholecystectomy who had an undetected gallbladder adenocarcinoma before surgery, and the frequency rises if a gallbladder perforation happens during the procedure.

With advantages in terms of hospital stay and postoperative problems, laparoscopic colonic surgery is both feasible and substantially equivalent to open surgery [1]. The COLOR II trial's three-year follow-up data were released in 2013. A total of 1103 individuals were chosen at random to have open or laparoscopic surgery in 30 centers. The patients in the laparoscopic group, according to the authors, saw a reduction in blood loss, a quicker onset of bowel motions, and a shorter hospital stay. These differences were statistically significant. The quality of the specimens did not differ. There were comparable rates of postoperative morbidity and mortality. Although hospitals with prior experience doing laparoscopic colorectal surgery were chosen for the surgical teams, the conversion rate was just 17% [28]. There can be multiple complications after laparoscopic surgeries. This article aims to focus on the various complications originating from the port site.

Port-Site Hernia

In gynecological surgery, Fear's 1968 paper was the first in the scientific literature to describe a port-site hernia (PSH) [29] and was acknowledged by Tonouchi et al. [30]. Even though this problem has long been known, the number of patients receiving this kind of treatment is increasing, highlighting its importance. There have been reports of PSH incidences ranging from 0.14% to 22% in a variety of laparoscopic operations. PSH can cause serious side effects such as intestinal blockage, strangling, and perforation, in addition to pain [4]. Ki et al. conducted a retrospective assessment of 18 patients who had umbilical PSH following laparoscopic abdominal surgery. The inter-recti distance (IRD) was calculated for each patient using preoperative computed tomography (CT) analysis. Additionally, measurements and analyses were made of trocar size, wound infection, body mass index (BMI), port extension, suture materials, and pre-existing co-morbidities. He observed that umbilical PSH was linked to the port incision's extension. Diastasis recti (DR) was present in 10 out of 18 patients with umbilical PSH (56%), before their initial laparoscopic procedure. Sarcopenia was present in nine (50%) individuals. In addition, four of the five recurrences had DR. Thus, the author concluded that sarcopenia and port extension were risk factors for umbilical PSH. Additionally, DR may be a potential risk factor for the development and recurrence of umbilical PSH. Before scheduling a laparoscopic procedure, doctors should be alerted if DR is present using diagnostic imaging. If umbilical PSH and DR are linked, we must address the treatment of both disorders simultaneously [6].

Bunting presented the results of a comprehensive literature search from 1995 to 2010 to track down any instances of port-site, trocar-site, or incisional hernia following laparoscopic cholecystectomy. 1.7% was the overall frequency of port-site hernias (range: 0.3% to 5.4). The most significant variables included advanced age, greater BMI, a prior hernia, trocar diameter and design, longer surgical duration, and an extension of the gallbladder extraction port site. The umbilical port was most frequently linked to incisional hernia, with the epigastrium and left and right hypochondrium coming next. Hernias have been associated with several medical conditions, such as diabetes mellitus, acquired immune deficiency syndrome, renal failure, and chronic obstructive pulmonary disease. It is believed that prolonged port manipulation and reinsertion, longer surgical times, failure to close the fascial defect, and wound infection effectively are responsible for the development of PSH [4]. Though it has not been generally adopted, PSH has been classified into three types: early (dehiscence of fascial planes and peritoneum), late (dehiscence of the fascial plane with intact peritoneal hernia sac), and special (dehiscence of the entire abdominal wall) [30]. Nassar et al. discovered that a preponderance of umbilical or paraumbilical defects predated laparoscopic cholecystectomy in 12% of patients, 83.7% of whom did not exhibit any symptoms. A preexisting hernia with fascial closure at the time of surgery was present in 25% of patients, of whom 1.8% developed incisional PSH. He also claimed that a higher prevalence of hernias seems to be linked to the male gender [7].

Using a Berci fascial closure instrument (suture retrieval needle) versus conventional suture closure of the umbilical fascia was examined in a single randomized experiment with 100 patients. In both groups, no patient experienced PSH. However, this study's sample size was too limited to have any practical significance [8]. In one facility, fascial closure of trocar sites is accomplished with a Deschamps ligature needle. All post locations, including 5-mm ports and the final port, can be closed with this manually operated, reusable blunt-tipped tool. There have been no reports of PSH in their 1400 laparoscopic operation series [31]. A case series of 54 patients who underwent surgery in two surgical centers for port-site incisional hernias was published by Lambertz et al. Depending on the surgical technique used for port-site hernia repair (mesh repair group, n=13 vs. suture alone group, n=41), their data were gathered and retrospectively analyzed. 96% of patients experienced a port-site incisional hernia following the application of trocars with a diameter of 10 mm or more. Compared to patients in the suture-only group, individuals treated with mesh repair had considerably higher BMI and significantly higher rates of heart illnesses. The group that underwent mesh repair had a substantially bigger mean fascial defect size (31 ± 24 mm vs. 24 ± 32 mm; p=0.007), while the group that underwent mesh repair had a significantly longer mean operation duration (83 ± 47 min vs. 40 ± 28 min; p<0.001). Between the study groups, there were no significant changes in the mean hospital stay (3 ± 4 days; p=0.057) or the hernia recurrence rate (9%; p=0.653) [9].

As documented in a case study by Tobe et al., on the 11th postoperative day following a radical prostatectomy with robotic assistance, a 73-year-old man experienced significant abdominal pain and nausea. Dilated small bowel loops were discovered using computed tomography images. Although adhesive ileus was first thought to be the cause, his symptoms worsened. A laparotomy was therefore carried out. Upon reopening the camera port wound, it was discovered that the small intestine and healed fascia were imprisoned within the peritoneal defects. These results aligned with the diagnosis of Richter's hernia [10]. In a case study published by Robicsek et al., a male patient, age 71, was diagnosed with an irreducible hernia at a port site from a previous laparoscopic operation when he arrived with a palpable, non-reducible right lateral periumbilical mass. Before this, he had bilateral inguinal hernia repairs performed laparoscopically using the 10 mm right lateral periumbilical port site defect within the musculoaponeurotic abdominal wall. Intraoperatively, an incarcerated appendix was found as content [11]. One of the issues for morbidly obese women is difficulty closing their ports, which results in insufficient rectus suturing and a hernia at the port site. A 59-year-old woman with severe obesity received a total laparoscopic hysterectomy, bilateral salpingo-oophorectomy, and pelvic lymph node dissection for endometrial cancer, according to a case report published in Vineet et al. The intraoperative phase was uneventful. She experienced acute obstruction in the postoperative phase as a result of a small bowel port-site herniation, which was not discovered until postoperative day 5. Due to the non-resolving obstruction, a CT scan was performed, and the results showed that a tiny intestinal loop had herniated through the umbilical port. Under local anesthesia, an immediate repair was necessitated. In the same instance, the rectus sheath was closed. After that, the patient recovered quickly; three days later, she was discharged. Hence, it was concluded that rectus sheath closure must be performed for all ports with a diameter of 10 mm or more. In postoperative obese women with unresolved gastrointestinal problems, there ought to be a low threshold for obtaining cross-sectional imaging [12].

Port-Site Infection

The port site infection (PSI) is one avoidable adverse effect of laparoscopic surgery (LS). The benefits of LS are undermined by PSI, which makes the patient anxious about the persistent infection and loses faith in the operative surgeon. Morbidity, length of stay in the hospital, and patient’s financial loss significantly rise. The ultimate goal of Minimal access surgery (MAS), which is to attain the highest level of cosmesis, is compromised, and patients' quality of life is negatively impacted. Because of the early discharge and daycare circumstances, active surveillance for PSIs in LS is still challenging [13]. Due to reduced incision length, the incidence of surgical site infections (SSI) following elective laparoscopic cholecystectomy is lower than that following open cholecystectomy. According to reports, the umbilical PSI rate in LS is 8%, with 89% of infections following laparoscopic cholecystectomy and 11% following laparoscopic appendectomy [32]. Lilani et al. noted a substantial rise in the incidence of SSIs with a preoperative stay longer than two days. Additionally, he stated that there was no infection rate in surgeries lasting less than 30 minutes. A substantial rise in SSIs was observed for surgeries lasting two hours or more [14]. Patients who have a history of diabetes, malnourishment, prolonged preoperative hospital stays, preoperative *Staphylococcus aureus* colonization of the nares, perioperative blood transfusions, and tobacco or steroid use are more likely to have PSI. Prophylactic antibiotics, obesity, and drains have no bearing on the incidence of SSIs after laparoscopic cholecystectomy [33,34].

PSIs are more prevalent in the umbilical port; the port used for removing the specimen may impact the infection rate. The infected material should be evacuated in an endo bag to avoid unintentional leaking of contents or occult malignant cells. A basic surgical glove can be used to create an improvised endobag that is inexpensive, easily obtainable, and disposable [35]. Over the course of a year, 675 patients with postoperative PSI following elective laparoscopic cholecystectomy for symptomatic cholelithiasis were included in prospective research by Mir et al. Culture swabs were sent to the microbiology lab from port locations exhibiting PSI symptoms. The antibiotic susceptibility test was performed on the positive swab cultures. Following an elective laparoscopic cholecystectomy, 6.7% of patients experienced PSI. Pseudomonas is the most common organism causing PSI, accounting for 19 (42.2%) cases. The majority of the isolates' strains were resistant to common antibiotics; for example, pseudomonas was found to be 100% resistant to ampicillin + sulbactam + ceftriaxone, but it was susceptible to imipenem, amikacin, and vancomycin in 89.47%, 57%, and 52.63% of cases, respectively [15]. The surgical technique and pathogenic organisms that cause surgical site infections vary. Clean surgical wounds typically include *S. aureus*, which may originate from the patient's local flora or an external source. Clean-contaminated, contaminated, and dirty surgical wounds can develop polymicrobial infections that resemble the target organ's indigenous flora. *S. aureus* was shown to be the most frequently isolated bacteria in superficial SSIs in both open surgical procedures and MAS, according to Kownhar et al. After analyzing the SSIs, they discovered that several common bacteria had been isolated, including Pseudomonas aeruginosa (37%) and *S. aureus* (37%). *Klebsiella pneumonia* (8%), Acinetobacter spp. (3.2%), Proteus spp. (4.8%), *Escherichia coli* (4.8%), *Citrobacter freundii* (1.6%), *Edwardsiella tarda* (1.6%), and *Enterococcus faecalis* (1.6%) were the next most common bacteria. Regardless of the surgical technique, Klebsiella sp. is the most frequent pathogenic organism in deep surgical site infections. Treatment with appropriate antibiotics after antibiotic sensitivity testing is the standard of care [36].

Non-tuberculous mycobacteria can contaminate medical equipment and can be found in soil and water from various sources. The most frequent reason for PSI with atypical mycobacteria is a breach of the laparoscopic instrument sterilization regimen. Due to the heat-sensitive outer insulating sheath on most laparoscopic equipment, infection with atypical mycobacteria is typically restricted to the laparoscopic procedure [13]. Furthermore, because most laparoscopic devices have several joints and fissures, tissue and blood can accumulate there. Regular device use without proper cleaning could lead to microbial infection, including atypical mycobacteria. There is no agreement on how to handle PSIs containing atypical mycobacteria. They are not well responsive to first-line antitubercular medication therapy. Promising outcomes have been observed when second-line antitubercular medications, such as macrolides (clarithromycin), quinolones (ciprofloxacin), tetracyclines (doxycycline), and aminoglycosides (amikacin and tobramycin), are taken in different combinations. The sole class of antimicrobials efficacious against *Mycobacterium chelonae* and *M. abscessus* are macrolides, which include clarithromycin. Because of a mutation in the bacterial wall's porin channels, which allow antibiotic molecules to enter for antibacterial activity, the *M. fortium chelonae* complex has demonstrated antibiotic resistance. Since it was shown to be effective against *M. chelonae*, linezolid has been used to treat patients either on its own or in combination with other medications [37,38].

In their review article, Sasmal et al. outline the ten commandments for preventing PSI: (i) utilization of disposable trocars as well as instruments; (ii) employing autoclavable laparoscopic hand instruments; (3) utilization of instruments with good ergonomics, limited joints, and facilities for proper cleaning of the debris collected in their crevices; (4) the most effective approach for proper cleaning of the instrument is ultrasonic technology. Utilizing autoclaved water to clean the instruments following disassembly; (5) adhering to the recommended concentration, contact duration, and usage cycles when sterilizing instruments with liquid sterilizers; (6) sterilizing instruments using ethylene oxide or a plasma sterilizer between surgeries; (7) refrain from sharing instruments between departments, such as utilizing equipment for gynecological or urological procedures; (8) prevent bile or gut content from spilling into the operating room or the port site; (9) use non-porous specimen retrieval bags for recovering the specimen; and (10) thoroughly clean and irrigate the port site before closing the wound [13].

Port-Site Hydatid Cyst

The causative agent of hepatic hydatid disease is *Echinococcus granulosus*. In humans, the liver carries 50-75% of the cysts, the lungs hold 25%, and the vascular system possesses 5-10% of the cysts. The third and fourth decades are the age groups most frequently impacted. Patients with hepatic hydatid disease may exhibit no symptoms at all or a combination of symptoms, such as jaundice, urticaria, malaise, fever, anorexia, hepatomegaly, and cough. The most common presenting types are mass and abdominal pain [39]. The first report of liver hydatid cysts being treated laparoscopically was published by Bickel et al. Shortly after, Khoury et al. reported the first report of anaphylactic shock complicating laparoscopic treatment of hydatid cysts of the liver [40]. A disproportionate concern about anaphylaxis appeared to be discouraging surgeons from using minimal access procedures to treat hydatid cysts more frequently. But nowadays, the laparoscopic route seems feasible and efficient for operating on hydatid cysts [41].

According to a case report published in 2022 by Aggarwal et al., a 17-year-old boy presented with a giant exophytic cyst originating from the left kidney's lower pole (BOSNIAK-1). The cyst was removed via laparoscopy. Histopathology determined it to be a hydatid cyst. After two years, he experienced pain for two days and an umbilical port site swelling for six months. An operation was performed to treat what was thought to be a strangulated incisional hernia at the port site. During the procedure, the hernia was fixed, and a port site cyst was removed, which was intact. Remarkable characteristics of a hydatid cyst were seen in the histopathology [16]. Another instance of a 40-year-old female patient who had previously undergone laparoscopic hepatic hydatid cystectomy and had a recurrence of hepatic hydatid cysts with port-site anterior abdominal wall hydatid cysts was described by Swamy et al. in 2021. ­­Hydatid cysts near the port site are an uncommon consequence of laparoscopic hydatid cystotomy; nonetheless, they can develop when scolices become lodged there during the removal of a daughter cyst during a laparoscopy. Excision is the treatment of choice in these cases, followed by anti-helminthic drugs [17].

Port-Site Tuberculosis

Port-site tuberculosis is a rare complication after a laparoscopic procedure. In developing nations like India, where the proportion of incidence of tuberculosis per year is the greatest in the world, port-site tuberculosis takes critical importance. Although endogenous infections can also occur at the port site, they are more frequently exogenous. Inadequate instrument sanitation and cleaning with tap water contaminated with resistant atypical mycobacteria before immersing them in glutaraldehyde solution are examples of exogenous transmission mechanisms [18]. Atypical Mycobacteria, like *M. chelonei*, have also been linked to a case of port-site infection following laparoscopic surgeries, in addition to *M. tuberculosis*. Mycobacteria may withstand glutaraldehyde's high-level chemical disinfection, typically used to sanitize laparoscopic equipment [19]. A discharging sinus at the epigastric port site developed in a young female patient following laparoscopic cholecystectomy, as reported in a case report by Faridi et al. Debridement and wound closure had been attempted three times previously, and there was a recurrence each time [20]. Once the sinus tract was removed following the sinogram, the histology revealed characteristics typical of tuberculosis. Following three months of follow-up, the patient on anti-tubercular therapy had not experienced any recurrence [42].

Cunnigaiper and Venkatraman [19] published another case report. An infertility evaluation was underway for a thirty-year-old woman. Her menstrual history was unremarkable, and her physical examination revealed nothing unusual. Her tubal patency had been evaluated during a laparoscopy. During the laparoscopy, there was very little fluid in the Douglas pouch and a few dispersed tubercles in the peritoneum. The fluid tested positive for tuberculosis by polymerase chain reaction, and a granulomatous lesion was discovered during the tubercles' biopsy. After that, she began antituberculous treatment (ATT). She experienced an abscess at the location of the umbilical port one month later. After draining the abscess, no organisms grew in the pus culture. She then experienced another abscess, which spontaneously ruptured to produce a sinus. After the sinus was opened and a biopsy of the wall was performed, tuberculosis was found. Following the patient's initiation of a modified ATT regimen, the wound healed completely and without recurrence [19]. A patient with non-healing sinuses at the epigastric and umbilical port sites manifested six weeks following laparoscopic cholecystectomy, according to a case report published by Jagdish et al. [18]. It was revealed to be extraperitoneal sinuses by a sinogram. *M. tuberculosis* was detected in a culture, and an edge biopsy of the lesion showed the existence of caseating granulomas. The patient underwent a nine-month course of ATT medication and a complete surgical excision of the sinuses. By secondary intention, the wound healed well, leaving scars at the umbilical and epigastric ports [19].

The most typical manifestation for these patients is usually a non-healing wound at the port site. Due to its association with maximum handling during surgery, the epigastric port site (in cases of lap cholecystectomy) or specimen retrieval site (in other laparoscopic procedures) is typically involved [18]. Pus culture and sensitivity tests rule out primary PSI or related secondary infections in mycobacterial infections. ZN staining can also directly demonstrate AFB. The polymerase chain reaction is beneficial in such isolated cases because it has a high negative predictive value for demonstrating mycobacterial DNA. Trace determination can be achieved by X-ray sinography and methylene blue dye used intraoperatively. Patients with such a presentation may be treated with ATT based on high suspicion or when a biopsy from the port site confirms the diagnosis. The patient's wound may heal with ATT alone or with ATT and sinus tract excision together [19,42].

Port Site Endometriosis

The existence of ectopic endometrial tissue outside the uterine cavity lining is known as endometriosis. The ovaries, cul-de-sac, and fallopian tubes are the pelvic locations where it most frequently occurs. Still, it can also be linked to the lungs, intestine, ureter, brain, and abdominal wall. Scar endometriosis, another name for abdominal wall endometriosis, is a very uncommon condition that primarily affects surgical scar sites. While most cases of scar endometriosis occur following cesarean sections, it can also happen following hysterectomy, episiotomy, and laparoscopic trocar port tracts. While uncommon, a doctor should rule out endometriosis if there is a painful mass in the surgical site, as the trocar site was discovered in a reproductive-age woman who has had pelvic or obstetric surgery in the past [21,22]. Many theories have been put forth, such as cellular immunity, coelomic metaplasia, implantation or retrograde menstruation, vascular and lymphatic metastases, dissemination, and direct transplantation. However, the exact etiopathogenesis of endometriosis is still up for debate. A scar endometrioma that develops after a caesarian delivery, hysterectomy, appendectomy, laparoscopic trocar tract, or episiotomy is most likely caused by direct transplantation. A trocar port site endometrioma may arise from direct contact between the excised lesion and the port tract or by peritoneal seeding of cells due to pneumoperitoneum [21,43].

Due to the concurrent presence of asymptomatic endometriosis in a portion of these patients, the widespread use of laparoscopic approaches by gynecologists for ovarian cyst excision, tubal ligation, and exploration to determine the cause of pelvic pain can result in the development of trocar site endometriosis [21,44]. In 1990, Denton et al. documented the initial instance of endometriosis at the trocar location [44]. Port-site endometriosis is typically diagnosed based on a patient's medical history and physical examination. In patients with the typical presentation of a palpable mass, cyclic pain, and a prior port site scar, this diagnosis is typically not difficult to make. This is particularly true in cases of gynecologic procedures that expose the uterine cavity or treat pelvic endometriosis [43]. While helpful, CT, ultrasonography (USG), and magnetic resonance imaging (MRI) are unable to offer a conclusive preoperative diagnosis. When women have abdominal wall masses linked to port-site endometriosis, ultrasound-guided FNAC (fine-needle aspiration cytology) is a quick and precise diagnostic method that helps rule out cancer and decide on the best course of action [23].

Port-site endometriosis has a broad differential diagnosis that is frequently mistaken with other pathologic disorders, including sebaceous cysts, desmoid tumors, suture granulomas, abscesses, soft-tissue sarcomas, lipomas, and metastatic tumors. Consequently, it is necessary to validate the pathologic diagnosis of endometriosis. Even when the disease recurs, the preferred course of treatment for scar endometriosis is extensive local excision of the lesion with negative margins. In addition, nonsteroidal anti-inflammatory drugs, oral contraceptives, gonadotropin-releasing hormone analogs, aromatase inhibitors, and radiofrequency ablation therapy are utilized in the medical management of port-site endometriosis [43]. Precautions such as collecting specimens from the abdominal cavity in endo bags, cleaning the incision sites, and carefully closing the abdomen cavity in patients who have a history of endometriosis or in whom pelvic endometriosis was discovered during laparoscopy can prevent the development of trocar site endometriosis [21]. A rare occurrence of port-site endometrioma appearing as an umbilical enlargement was reported by Siddiqui et al. [22]. Six months after undergoing laparoscopy for pelvic endometriosis, the patient complained of cyclical menstrual pain accompanied by swelling at the scar close to her umbilical port. A well-defined lesion in the umbilicus was discovered by an ultrasound scan, and an MRI ruled out secondary pathology. She had an exploration of the scar and an excision of the endometrioma. Histopathology confirmed the diagnosis [22].

Port-Site Metastasis

Port-site metastasis (PSM) is the term for tumor-cell implantation at the trocar insertion site after a malignant tumor is removed laparoscopically. PSM has been reported in 1-2% of laparoscopic gynecologic surgical procedures. "Solitary port-site metastasis," also known as "iPSM," is the term used to describe tumor recurrence at trocar sites in the absence of simultaneous metastasis. Conversely, PSM that disperses concurrently to other regions ("non-isolated PSM") is usually considered part of systemic recurrence. Although iPSM treatment is debatable, PSM with multiple metastases should be managed according to the standard guidelines for treating a systematic recurrence. For iPSM therapy, three options are available: irradiation alone, adjuvant chemotherapy, and radiation plus surgical excision [24,45]. A few potential mechanisms for cell implantation at the port site include embolization of exfoliated cells during tumor dissection or hematogenous spread, air turbulence during extended laparoscopic operations, and direct implantation onto the wound during forced, unprotected organ/tissue retrieval or from contaminated surgical instruments during tumor dissection. Nonetheless, the triggering mechanism is likely essentially multifaceted [45,46].

Several precautionary measures can be used to prevent PSM. When retrieving tissue, protective bags (also known as endo bags) and wound protectors are used. The pneumoperitoneum should not be removed while trocars are in place. The solid tumor and the port site are prevented from coming into direct contact. During the surgical or operative procedure, try not to manipulate the tumor bulk too much [46]. Heparin- or cytocidal-based peritoneal washing is used to stop the adherence of free cells. The usefulness of taurolidine (which has anti-adhesion activity and reduces proangiogenic factors) and povidone-iodine [47] is assessed. Compared to standard CO_2_ pneumoperitoneum, hyperthermic and humidified CO_2_ insufflation improves the prognosis for patients with malignant tumors undergoing laparoscopic procedures [48]. Excision of port sites appears to be a viable strategy to prevent PSM, given the substantial incidence of PSM. Still, doubts remain about the advantages of port-site resection. Lago et al. [25] assessed the effect of post-laparotomy port-site resection on the oncologic result of advanced ovarian cancer. They discovered that port-site excision increased the risk of wound complications and had no beneficial effect on survival [25].

Agarwala et al. conducted a retrospective analysis and found that eleven out of the 13 patients with solitary PSM had wide excisions. While seven patients underwent neoadjuvant chemotherapy (NACT) and two underwent neoadjuvant chemoradiation (NACTRT) before attempting resection, six patients underwent upfront resection. Overall survival (OS) was 37 months, and post-PSM disease-free survival (DFS) was 20 months, with a median follow-up of 22 months. As a result, they concluded that treating individuals with isolated PSM would benefit from careful selection combined with an aggressive treatment plan [26]. According to Z'graggen et al., patients undergoing laparoscopy or laparoscopic cholecystectomy who have an undiagnosed gallbladder adenocarcinoma have a high incidence of recurrences at the port site. This incidence rises if a gallbladder perforation occurs during the procedure [27]. The management of these PSMs involves histopathological biopsy and radiological imaging (CT/positron emission tomography [PET] scan), followed by wide local excision of the PSM. Adjuvant therapy in terms of chemotherapy or radiation can be given upon discussion with a medical oncologist [26,48]. 

Limitations

This narrative review has several limitations. First, the potential for subjective bias is inherent in the selection and interpretation of literature, as the search strategy was not exhaustive, and relevant studies may have been inadvertently missed. Due to funding limitations and limited resources, we have only examined PubMed and Google Scholar. We were not able to access the majority of other databases because they were paid for. The conclusions drawn are based on a selective set of studies, which may affect the generalizability of the findings. For the same reasons, a narrative rather than a systematic review was conducted. Additionally, while the review aims to provide a comprehensive overview, it may not cover all aspects of the topic in depth due to the narrative approach. Unlike most studies on individual port site complications, our study is a comprehensive review covering all possible diverse complications around the laparoscopic port site, making it unsuitable for any statistical analysis.

## Conclusions

The use of laparoscopic surgery as the norm for many operations has gained widespread acceptance. Various complications occur at the port site, such as port-site hernia, infection, hydatid cysts, tuberculosis, endometriosis, and metastatic deposits. Prevention is better than cure. Smaller trocars and meticulous rectus sheath defect closure at the end of surgery can prevent port-site hernias. Employing stringent sterilization techniques and strict aseptic precautions, such as using non-porous specimen retrieval bags to recover the specimen, can prevent the rest of the port-site complications.
